# Decreased RXRα is Associated with Increased β-Catenin/TCF4 in ^56^Fe-Induced Intestinal Tumors

**DOI:** 10.3389/fonc.2015.00218

**Published:** 2015-10-08

**Authors:** Shubhankar Suman, Santosh Kumar, Albert J. Fornace, Kamal Datta

**Affiliations:** ^1^Department of Biochemistry and Molecular and Cellular Biology, Lombardi Comprehensive Cancer Center, Georgetown University, Washington, DC, USA; ^2^Center of Excellence in Genomic Medicine Research (CEGMR), King Abdulaziz University, Jeddah, Saudi Arabia

**Keywords:** APC^Min/+^, intestinal tumor, space radiation, heavy ion radiation, tumorigenesis, proteasome, β-catenin

## Abstract

Although it is known that accumulation of oncogenic β-catenin is critical for intestinal tumorigenesis, the underlying mechanisms have not yet been fully explored. Post-translational β-catenin level is regulated via the adenomatous polyposis coli (APC)-dependent as well as the APC-independent ubiquitin–proteasome pathway (UPP). Employing an APC-mutant mouse model (APC^Min/+^) the present study aimed to investigate the status of RXRα, an APC-independent factor involved in targeting β-catenin to UPP for degradation, in tumor-bearing and tumor-free areas of intestine after exposure to energetic ^56^Fe ions. APC^Min/+^ mice were exposed to energetic ^56^Fe ions (4 or 1.6 Gy) and intestinal tumor samples and tumor-free normal intestinal samples were collected 100–110 days after exposure. The status of TCF4, β-catenin, cyclin D1, and RXRα was examined using immunohistochemistry and immunoblots. We observed increased accumulation of the transcription factor TCF4 and its co-activator β-catenin as well as their downstream oncogenic target protein cyclin-D1 in ^56^Fe ion-induced intestinal tumors. Further, decreased expression of RXRα in tumors as well as in adjacent normal epithelium was indicative of perturbations in β-catenin proteasomal-targeting machinery. This indicates that decreased UPP targeting of β-catenin due to downregulation of RXRα can contribute to further accumulation of β-catenin and to ^56^Fe-induced tumorigenesis.

## Introduction

Heavy ion charged particle (HZE) radiation, such as ^56^Fe ions, is prevalent in deep space, and is a major concern for astronauts’ health (1). Recently, using APC^Min/+^ mice, a well-accepted mouse model for human colorectal cancer (CRC), we found increased risk of CRC development accompanied by increased nuclear accumulation of oncogenic β-catenin and activation of its downstream signaling after exposure to ^56^Fe ions (2–5). However, the mechanisms behind the accumulation of oncogenic β-catenin are not yet fully understood.

Cellular levels of free β-catenin are tightly regulated via the ubiquitin–proteasome pathway (UPP). Targeting of β-catenin to the proteasome and its subsequent degradation involves two adenomatous polyposis coli (APC)-dependent (i.e., APC/GSK3β/AXIN and APC/Siah1) and one APC-independent (RXRα-mediated) mechanisms (6). In gastrointestinal (GI) tumors, genes involved in APC-dependent (APC, Siah1, and Axin) targeting of β-catenin are often mutated (7–11), and similarly in APC^Min/+^ mice, tumor formation is mostly driven through inactivation of the wild type APC allele (12). Thus, APC-dependent proteasomal targeting of β-catenin is eventually disabled in these tumors. In the absence of proteasomal targeting, β-catenin accumulates and interacts with T-cell factor transcription factors (TCF4) in the nucleus leading to activation of oncogenic signaling pathways (13). In view of the known perturbations in APC-dependent proteasomal targeting of β-catenin early in the GI tumorigenesis process, only the APC-independent (RXRα-dependent) pathway would remain to control its accumulation. However, the status of the APC-independent proteasomal targeting of the β-catenin in heavy ion radiation-induced intestinal tumors has not been explored. In this study, using the APC^Min/+^ intestinal tumor mouse model (14), we demonstrated downregulation of RXRα expression, which may complement the disabled APC-dependent proteasomal degradation pathway to increase β-catenin accumulation in ^56^Fe-induced tumors. Downregulation of RXRα observed in this study could potentially play a crucial role in heavy ion radiation-induced increased risk of intestinal tumorigenesis and would warrant further investigation.

## Materials and Methods

### Mice and genotyping

Male APC^Min/+^ mice (The Jackson Laboratory, Bar Harbor, ME, USA) were bred with female C57BL/6J mice at the Georgetown University (GU)’s animal facility. Genotyping using tail DNA samples were done using reverse-transcription polymerase chain reaction (RT-PCR) to identify heterozygous offspring as per the Jackson Laboratory protocol. The mouse colony was maintained on standard certified rodent diet and filtered water in a humidity and temperature-controlled room with 12 h dark/light cycle. All experimental procedures were performed in compliance with the protocols approved by the Institutional Animal Care and Use Committee (IACUC) at GU and Brookhaven National Laboratory (BNL). Both the facilities are Association for Assessment and Accreditation of Laboratory and Animal Care International (AAALACI) accredited facilities and we followed The Guide for the Care and Use of Laboratory Animals.

### Irradiation and sample collection

APC^Min/+^ female mice (6–8-weeks old) were placed in well-ventilated transparent plastic boxes (1 mouse/box) allowing easy movement and irradiated with 4 or 1.6 Gy whole body ^56^Fe radiation (energy: 1000 MeV/n; LET: 148 keV/μm; dose rate: 1 Gy/min) at the NASA Space Radiation Laboratory (NSRL) at BNL. These two doses were used in our previously published tumorigenesis experiments and samples collected during that study were used for molecular analysis in this study. For ^56^Fe exposure, both control and treatment groups were shipped to BNL for irradiation and brought back to GU after irradiation in a temperature-controlled vehicle for a same day delivery to minimize stress to the animals. Age-matched ^56^Fe-irradiated and control mice were euthanized by CO_2_ asphyxiation between 100 and 110 days after radiation exposure. The small intestinal tract was surgically removed, washed with phosphate-buffered saline (PBS), and cut open longitudinally at room temperature. A dissecting scope (Leica MZ6, Buffalo Grove, IL, USA) was used to visualize and dissect tumors, which were then flash frozen in liquid nitrogen and stored at −80°C for further use. Also, intestinal samples (~3 cm) with tumor-bearing and surrounding tumor-free area were fixed overnight in 10% buffered formalin, embedded in paraffin, and 4 μm-thick sections were obtained for immunohistochemistry staining.

### Immunohistochemistry

Intestinal sections (*n* = 5 mice per group) were used for immunohistochemistry with a protocol described earlier (3). Briefly, immunostaining for active-β-catenin (Cat#05-665, Millipore, Billerica, MA, USA; dilution: 1:100), TCF4 (Cat#05-511, Millipore; dilution: 1:100), RXRα (Cat#sc-553, Santa Cruz Biotechnology, Dallas, TX, USA; dilution: 1:40), and cyclin D1 (Cat#04-1151; Millipore; dilution: 1:150) were performed by soaking slides in antigen retrieval citrate buffer (pH 6.0; Dako, Carpinteria, CA, USA) and heating at 100°C for 15 min in a microwave oven. Further, endogenous peroxidase activity was quenched using 3% hydrogen peroxide in methanol followed by incubation in blocking buffer (5% bovine serum albumin in PBS) for 30 min. After blocking sections were incubated overnight at 4°C with the respective primary antibody. Signal detection and color development was done using SuperPicture 3rd Gen IHC detection kit (Cat#87-9673; Invitrogen, Carlsbad, CA, USA). Sections were counterstained using hematoxylin and images were acquired using bright field microscopy at a magnification of 20×. At least 10 randomly chosen images from the tumor-bearing as well as from the tumor-free areas were acquired from each mouse and a representative image from each group is shown in the results. Images were analyzed using color deconvolution and image-based tool for counting nuclei (ITCN) plug-ins of ImageJ v1.45 software (National Institutes of Health, Bethesda, MD, USA). Quantification data were statistically analyzed using two-tailed paired Student’s *t*-test and difference between control and irradiated group was considered significant when *p*-value was <0.05. Error bars represent mean ± SEM.

### Immunoblots

Frozen intestinal tumor samples (*n* = 5 mice per group) were pooled and used for immunoblot analysis of RXRα level with a protocol described previously (3). Briefly, samples were homogenized in ice-cold lysis buffer, centrifuged, and supernatant collected. Protein was estimated in supernatant and equal amount of protein was used for sodium dodecyl sulfate-polyacrylamide gel electrophoresis (SDS-PAGE). Protein was transferred to PVDF membrane, incubated with RXRα antibody, and protein detected using horseradish peroxidase (HRP) conjugated secondary antibody and enhanced chemiluminescence (ECL) detection system (Cat# 34080, Thermo Fisher Scientific, Rockford, IL, USA) and representative images shown in the results.

## Results

### Increased β-catenin and TCF4 levels in ^56^Fe-induced intestinal tumor

**Figure 1:**
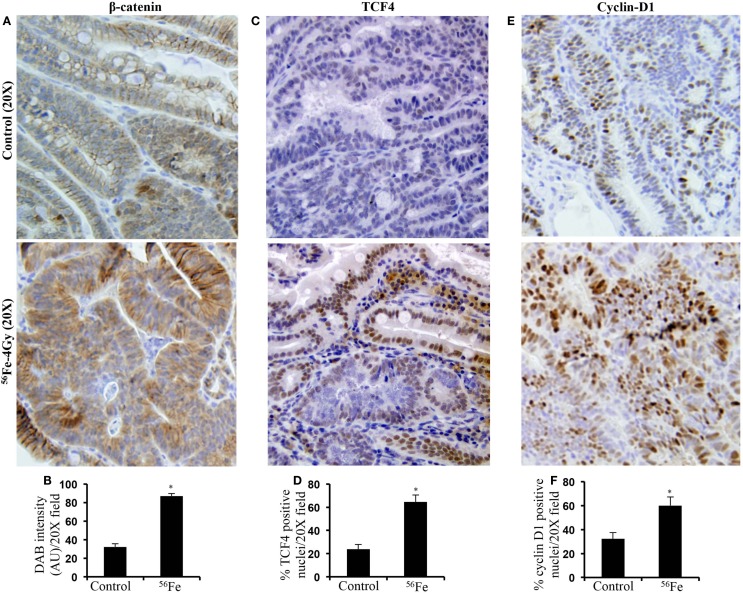
**Accumulation and activation of oncogenic β-catenin signaling in ^56^Fe-induced intestinal tumors compared to spontaneous tumors from sham-irradiated mice (control)**. **(A)** Immunohistochemical detection of active-β-catenin in ^56^Fe-induced intestinal tumors. **(B)** Quantification of β-catenin expression in intestinal tumors. **(C)** Immunohistochemical detection of β-catenin transcriptional regulator TCF4 in ^56^Fe-induced intestinal tumors. **(D)** Quantification of TCF4 positive nuclei in intestinal tumors. **(E)** Immunohistochemical detection of β-catenin/TCF4 oncogenic target cyclin-D1 in ^56^Fe-induced intestinal tumors. **(F)** Quantification of cyclin-D1 positive nuclei in intestinal tumors. Error bars represent mean ± SEM and *p* < 0.05 was considered significant. AU – Arbitrary Unit.

Intestinal tumors stained for β-catenin showed increased level in 4 Gy ^56^Fe-irradiated samples relative to control tumors from sham-irradiated mice (Figures 1A,B) and this is consistent with our previous results after 1.6 Gy ^56^Fe irradiation (3). Higher levels were also observed for TCF4 in ^56^Fe-irradiated intestinal tumors relative to controls (Figures 1C,D). Transcription factor TCF4 along with the transcriptional co-activator β-catenin are involved in transcribing pro-proliferative factors, such as cyclin D1, and increased cyclin D1 was observed in the current study (Figures 1E,F) as well.

### Reduced expression of RXRα in tumor-bearing and tumor-free areas of APC^Min/+^ mice after exposure to ^56^Fe radiation

**Figure 2:**
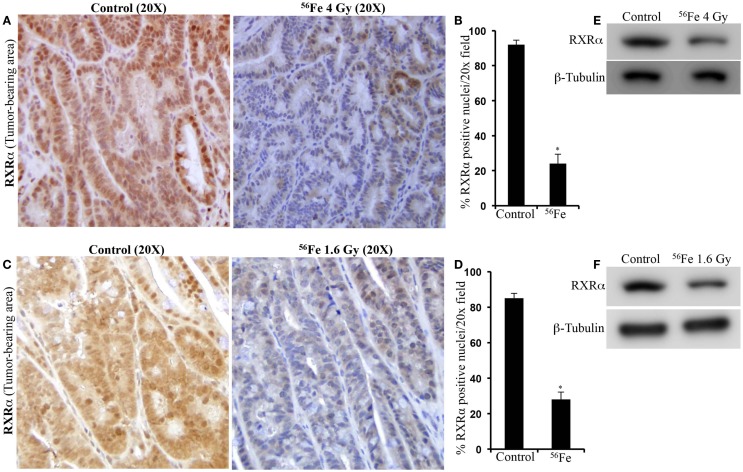
**Lower RXRα expression in ^56^Fe-induced intestinal tumors**. **(A)** Representative immunohistochemistry images showing expression of RXRα in spontaneous and 4 Gy ^56^Fe-induced intestinal tumors. **(B)** Quantification of RXRα in spontaneous and 4 Gy ^56^Fe-induced intestinal tumors. **(C)** Expression of RXRα in spontaneous and 1.6 Gy ^56^Fe-induced intestinal tumors. **(D)** Quantification of RXRα in spontaneous and 1.6 Gy ^56^Fe-induced intestinal tumors. **(E)** Immunoblots of RXRα in spontaneous and 4 Gy ^56^Fe-induced intestinal tumors. **(F)** Immunoblots of RXRα in spontaneous and 1.6 Gy ^56^Fe-induced intestinal tumors. Error bars represent mean ± SEM and *p* < 0.05 was considered significant.

**Figure 3:**
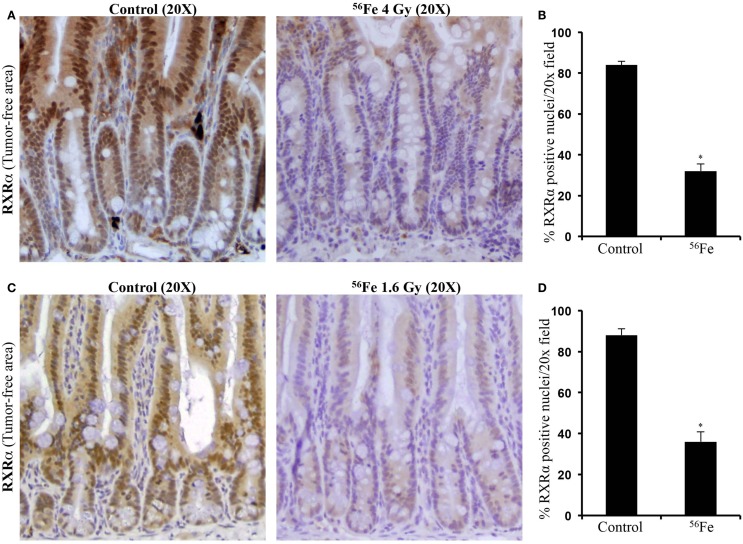
**Downregulation of RXRα was observed in tumor-free areas of intestinal samples from APC^Min/+^ mice**. **(A)** Decreased RXRα expression after 4 Gy ^56^Fe radiation. **(B)** Quantification of immunohistochemistry images showed significant decrease in RXRα after 4 Gy ^56^Fe. **(C)** Decreased RXRα expression after 1.6 Gy ^56^Fe radiation. **(D)** Quantification of immunohistochemistry images showed significant decrease in RXRα after 1.6 Gy ^56^Fe. Error bars represent mean ± SEM and *p* < 0.05 was considered significant.

Immunohistochemistry in tumor samples demonstrated that expression of RXRα was reduced after 4 Gy (Figure 2A). Quantification and statistical analysis of stained sections from five mice showed that RXRα was significantly lowered in ^56^Fe-irradiated tumors relative to sham-irradiated tumors (Figure 2B). Intestinal tumors from 1.6 Gy ^56^Fe-irradiated mice also showed decreased RXRα staining (Figure 2C) and quantification and statistical analysis showed that the staining in irradiated samples were significantly lower compared to controls (Figure 2D). However, quantification did not show significant difference in RXRα staining between two radiation doses. Immunoblots of 4 Gy (Figure 2E) and 1.6 Gy (Figure 2F) intestinal tumor samples also showed decreased RXRα. We also performed immunohistochemistry for RXRα on tumor-free intestinal sections from APC^Min/+^ mice exposed to either 1.6 or 4 Gy ^56^Fe ions. Staining of tumor-free intestinal section showed lower expression of RXRα after 4 Gy ^56^Fe relative to corresponding controls (Figure 3A). Decreased RXRα after 4 Gy ^56^Fe was statistically significant compared to sham-irradiated controls (Figure 3B). Conversely, we also observed downregulation of RXRα in 1.6 Gy irradiated samples compared to controls (Figure 3C) and quantification showed statistically significant difference between irradiated and sham-irradiated samples (Figure 3D).

## Discussion

The carcinogenic potential of ionizing radiation is well known and using animal models it has been established that high-LET heavy ion radiation has higher carcinogenic potential compared to low-LET radiation (15). Increased frequencies of site-specific cancer following heavy ion exposure have been reported in various rodent models with upregulation of oncogenic signaling mediated through genetic, epigenetic, and/or physiological changes (3, 15–17). Earlier studies conducted in APC^Min/+^ mice revealed increased tumor induction and a higher number of adenocarcinomas, which was associated with greater upregulation of β-catenin signaling after ^56^Fe exposure relative to γ radiation; this is indicative of perturbations in the molecular events upstream of β-catenin (3). The purpose of the current study was to develop mechanistic insight into greater tumorigenesis observed in our previous work in APC^Min/+^ mice after two doses of ^56^Fe radiation relative to γ radiation. While pathways can be investigated in the wild-type mice, they are resistant to intestinal tumorigenesis. Therefore, we used APC-mutant mice not only to quantitatively assess tumor frequency but also to understand molecular pathway alterations, which may have contributed to tumor development after radiation exposure. While we reported previously that two doses of ^56^Fe caused higher tumor frequency, we are yet to fully understand molecular characteristics of the tumors and tumor-adjacent normal tissues after ^56^Fe irradiation. To this end, the results presented in the current study explain in part potential underlying mechanisms contributing to increased tumor frequency after ^56^Fe irradiation. We have focused on the APC-independent mechanism of β-catenin degradation via UPP. In APC-deficient adenoma, accumulation of β-catenin complexed with nuclear TCF4 results in the increased expression of its oncogenic target genes, such as cyclin-D1 that promotes intestinal cell proliferation and polyp formation (18). In agreement with our published reports in APC^Min/+^ mice exposed to 1.6 Gy of ^56^Fe ion, the current study also observed similar activation of β-catenin at 4 Gy of ^56^Fe ion along with increased TCF4 and cyclin-D1. Significant loss of RXRα was evident in tumors as well as in tumor-free areas of intestine after ^56^Fe radiation and this could contribute to decreased proteasomal targeting of β-catenin, therefore enhancing cell survival and proliferation through β-catenin/TCF4 signaling. Notably, RXRα was downregulated in both the radiation doses tested suggesting that the effect is independent of radiation dose and that the lower dose may have a proportionately greater effect relative to the higher dose. We recognize that the mean absorbed doses of energetic ^56^Fe ions used in the current study are higher than the doses astronauts are expected to receive during prolonged space missions. These high doses of energetic ^56^Fe ions were used as a proof of principle in our initial studies for establishing the differential effects, quantitatively and qualitatively, of space compared to γ radiation.

Loss of the remaining wild type APC allele has often been implicated as the primary mechanism for increased β-catenin signaling leading to tumor development in APC^Min/+^ mice (12, 19, 20). In addition to APC, the β-catenin cellular level is also regulated through a direct proteasomal targeting mediated by RXRα (21) and downregulation of RXRα in human and rodent colonic tumors has been reported previously (22). Considering that protein turnover is critical for cellular homeostasis, availability of multiple independent pathways for protein degradation ensures that the potentially pro-carcinogenic β-catenin level remains within physiologic limits to limit cancer initiation and progression. Downregulation of RXRα in our model system may have played a role in ^56^Fe radiation-induced more aggressive tumorigenesis reported earlier (3). Apart from driving proteasomal degradation of β-catenin, RXRα also functions to suppress β-catenin-mediated upregulation of oncogenes through direct protein–protein interaction (23) in colon cancer cells. Thus, loss of RXRα expression could further stabilize β-catenin signaling in tumor cells, leading to greater cell proliferation and higher number of invasive cancers associated with ^56^Fe relative to γ radiation.

**Figure 4:**
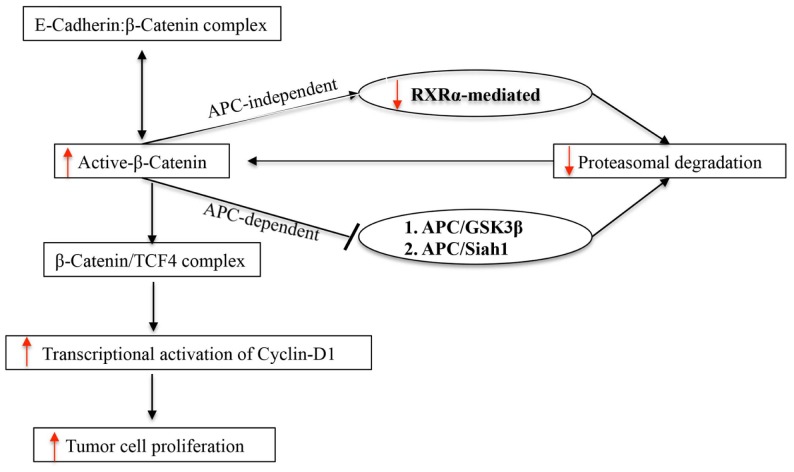
**Schematic representation of APC-dependent and APC-independent pathways of UPP involved in β-catenin degradation in APC^Min/+^ mice**.

Nuclear receptor RXRα is known to heterodimerize with a host of other nuclear receptors, such as the vitamin D receptor (VDR) and retinoid acid receptor (RAR), and is involved through transactivation of target genes, such as p21, in regulating normal growth and development (23, 24). Consequently, loss of RXRα is expected to cause disordered cellular proliferation, and indeed, downregulation of RXRα has been widely reported in a number of cancers including CRC (21–26). Our result demonstrates for the first time that RXRα is downregulated in tumor-free areas of APC^Min/+^ intestine ~100 days after radiation exposure. Considering that a significant number of intestinal adenomas has also been reported to arise without the loss of heterozygosity of the APC gene and these adenomas are often polyclonal (8, 20, 27), our results supports an APC-independent mechanism of β-catenin stabilization during ^56^Fe-irradiated tumorigenesis. We believe that decreased RXRα expression in tumor-free areas of the intestine may be a reflection of the RXRα status in other areas of the GI tract and that this molecular event may be preceding intestinal tumorigenesis in APC^Min/+^ mice. Furthermore, RXRα signaling is also linked to cellular redox regulation and it has been demonstrated that RXRα activation protects cell from oxidative stress and inhibition promotes ROS production (28, 29). Downregulation of RXRα observed ~100 days post-exposure in the current study aligns with our previous studies demonstrating chronic oxidative stress even 1 year after exposure to energetic ^56^Fe ions (30). Although we observed persistent oxidative stress after γ radiation, it was less pronounced relative to equitoxic doses of ^56^Fe radiation. Additionally, intestinal tumor frequency and grade was also higher after ^56^Fe relative to equitoxic doses of γ radiation. Considering that γ radiation responses were consistently lower relative to ^56^Fe, in the current study, we have analyzed and presented ^56^Fe-induced alterations of an alternate pathway involved in β-catenin regulation via RXRα. Our data, previous and current, demonstrate that effects of radiation on redox balance, carcinogenesis, and related molecular pathways are dependent on radiation quality and energy deposition characteristics. However, further in depth studies will be required to dissect the link between heavy ion radiation exposure and long-term molecular alterations, such as oxidative stress and RXRα downregulation. In summary, our results show that energetic heavy ion radiation is capable of lowering RXRα in tumor as well as non-tumor intestinal epithelial cells. Due to its roles in multiple cellular processes, continuous downregulation of RXRα, we believe, will have major ramifications for intestinal cellular homeostasis with implications for carcinogenesis including colorectal carcinogenesis (Figure 4).

## Author Contributions

Conceived and designed the experiments: SS and KD. Performed the experiments: SS and SK. Analyzed the data: SS, SK, and KD. Contributed reagents/materials/analysis tools: AF and KD. Wrote the paper: SS, AF, and KD. All authors read and approved this manuscript.

## Conflict of Interest Statement

The authors declare that the research was conducted in the absence of any commercial or financial relationships that could be construed as a potential conflict of interest.
